# *Cryptosporidium* Prevalence and Risk Factors among Mothers and Infants 0 to 6 Months in Rural and Semi-Rural Northwest Tanzania: A Prospective Cohort Study

**DOI:** 10.1371/journal.pntd.0003072

**Published:** 2014-10-02

**Authors:** Sarah H. Pedersen, Amanda L. Wilkinson, Aura Andreasen, David C. Warhurst, Safari M. Kinung'hi, Mark Urassa, Denna M. Mkwashapi, Jim Todd, John Changalucha, Joann M. McDermid

**Affiliations:** 1 Division of Nutritional Sciences, Cornell University, Ithaca, New York, United States of America; 2 Mwanza Intervention Trials Unit, London School of Hygiene and Tropical Medicine, Mwanza, Tanzania; 3 Department of Pathogen Molecular Biology, London School of Hygiene and Tropical Medicine, London, United Kingdom; 4 National Institute for Medical Research, Mwanza, Tanzania; 5 Department of Population Health, London School of Hygiene and Tropical Medicine, London, United Kingdom; Ege University, Turkey

## Abstract

**Background:**

*Cryptosporidium* epidemiology is poorly understood, but infection is suspected of contributing to childhood malnutrition and diarrhea-related mortality worldwide.

**Methods/Findings:**

A prospective cohort of 108 women and their infants in rural/semi-rural Tanzania were followed from delivery through six months. *Cryptosporidium* infection was determined in feces using modified Ziehl-Neelsen staining. Breastfeeding/infant feeding practices were queried and anthropometry measured. Maternal *Cryptosporidium* infection remained high throughout the study (monthly proportion = 44 to 63%). Infection did not differ during lactation or by HIV-serostatus, except that a greater proportion of HIV-positive mothers were infected at Month 1. Infant *Cryptosporidium* infection remained undetected until Month 2 and uncommon through Month 3 however, by Month 6, 33% of infants were infected. There were no differences in infant infection by HIV-exposure. Overall, exclusive breastfeeding (EBF) was limited, but as the proportion of infants exclusively breastfed declined from 32% at Month 1 to 4% at Month 6, infant infection increased from 0% at Month 1 to 33% at Month 6. Maternal *Cryptosporidium* infection was associated with increased odds of infant infection (unadjusted OR = 3.18, 95% CI 1.01 to 9.99), while maternal hand washing prior to infant feeding was counterintuitively also associated with increased odds of infant infection (adjusted OR = 5.02, 95% CI = 1.11 to 22.78).

**Conclusions:**

Both mothers and infants living in this setting suffer a high burden of *Cryptosporidium* infection, and the timing of first infant infection coincides with changes in breastfeeding practices. It is unknown whether this is due to breastfeeding practices reducing pathogen exposure through avoidance of contaminated food/water consumption; and/or breast milk providing important protective immune factors. Without a *Cryptosporidium* vaccine, and facing considerable diagnostic challenges and ineffective treatment in young infants, minimizing the overall environmental burden (e.g. contaminated water) and particularly, maternal *Cryptosporidium* infection burden as a means to protect against early infant infection needs prioritization.

## Author Summary

Early infancy and childhood *Cryptosporidium* infection is associated with poor nutritional status, stunted growth, and cognitive deficits, yet minimal research is available regarding the burden and risk factors worldwide. Since there is no vaccine available, and because diagnostic challenges exist and treatment for children younger than one year is ineffective, prevention of early infancy infection through a better understanding of basic epidemiology is critical. This study was designed to investigate symptomatic and clinically silent infection amongst HIV-seropositive and HIV-seronegative mothers and their infants in a longitudinal cohort, and to indentify potential risk factors. Findings indicate that infants are living in a *Cryptosporidium* environment as demonstrated by the chronically high level of maternal infection throughout the 6-month post-partum period. Despite this, infant infection prevalence remains low until six months of age when it dramatically rises. The increase in infant infection corresponds to a reduction in exclusive breastfeeding. As expected, maternal infection is associated with increased infant infection, but unexpectedly, so is maternal hand washing prior to infant feeding. Since prevention may indeed be the “best medicine” for infants, investigation of beneficial breastfeeding practices, protective correlates in breast milk, and ways to reduce the maternal and environmental *Cryptosporidium* burden are needed.

## Introduction

The World Health Organization reports that the most common diarrhea-causing protozoan parasite worldwide is *Cryptosporidium* [1], and a recent, large, multi-country investigation reported *Cryptosporidium* as the second most common pathogen indentified among care-seeking African and Asian infants 0 to 11 months [2]. The significance of this infection was underscored as this study revealed that infection was associated with a greater than two-fold increase in mortality of children 12 to 23 months [2]. Despite these indications of the potential global scope and impact of *Cryptosporidium* infection, a full understanding of the epidemiology of infant and early childhood infection remains limited due to logistical and methodological difficulties in conducting such research in impoverished high-burden urban and rural settings [2].

*Cryptosporidium* is a pathogen transmitted via the oral-fecal route from human (*C. hominis*) and animal (predominately *C. parvum*) reservoirs. Infection risk factors include a contaminated environment with elements such as: unsafe water, poor sanitation and hygiene, and close proximity to infected livestock, while severe clinical disease risk factors include: malnutrition and compromised immunity, particularly HIV-associated immunosuppression [1], [3]. Symptoms include: nausea, vomiting, voluminous and watery diarrhea, dehydration, abdominal discomfort, anorexia, fever, fatigue, and respiratory problems [4], [5], with chronic and life-threatening symptoms possible amongst immunocompromised individuals due to the increased duration and severity of illness [4]. However an unknown number of individuals experience asymptomatic *Cryptosporidium* infection [6]. This clinically silent infection may remain undetected and untreated and therefore may contribute to malnutrition, growth impairment, and long-term cognitive and functional deficits in infants and children [7], [8].

The primary aim of this research was to determine the prevalence of *Cryptosporidium* in young infants living in rural and semi-rural Tanzania by indentifying the timing of the first and subsequent *Cryptosporidium* events in both symptomatic and asymptomatic infections. Secondarily, we aimed to evaluate potential infant infection risk factors including: infant nutritional status, infant feeding practices, infant HIV-exposure, maternal nutritional status, maternal HIV infection, and, uniquely, maternal post-partum *Cryptosporidium* infection.

## Methods

This study was a prospective birth cohort enrolling newborns and their HIV-seropositive or –negative mothers living in the rural and semi-rural areas of Kisesa Ward (population 30,000) [9] in northwestern Tanzania. Pregnant women receiving antenatal care at Kisesa Health Centre (KHC), a Tanzanian government-administered, publically accessible primary care facility were recruited from March through December, 2012, a period that included both the dry and rainy seasons. Women gave birth between April, 2012 and January, 2013; the study follow-up appointments for mothers and infants were conducted between May, 2012 and July, 2013. Eligibility criteria were gestation <37 weeks at consent, singleton birth, known maternal HIV serostatus (screening with Determine HIV-1/2 [Inverness Medical], confirmation with Uni-Gold HIV-1/2 [Trinity Biotech]), maternal ability to speak and understand the local language of Kiswahili, and stated intention to reside within the clinic catchment at delivery and through six months post-partum. The study was advertised through health workers at KHC as well as rural government-run health dispensaries in the region. All HIV-positive women were receiving anti-retroviral treatment (ART) for their own care or for prevention of mother-to-child transmission by the time of delivery. Infants born to HIV-positive women were given nevirapine daily for six weeks and tested for HIV-infection by dried blood spot DNA-PCR at the regional hospital laboratory at the Month 3 follow-up visit. The study protocol was approved by the ethics review committees of the Tanzania National Health Research Ethics Review Committee and Cornell University. Written informed consent was obtained from mothers for themselves and on behalf of their infants at enrolment with verbal assent re-confirmed at follow-up.

**Figure 1:**
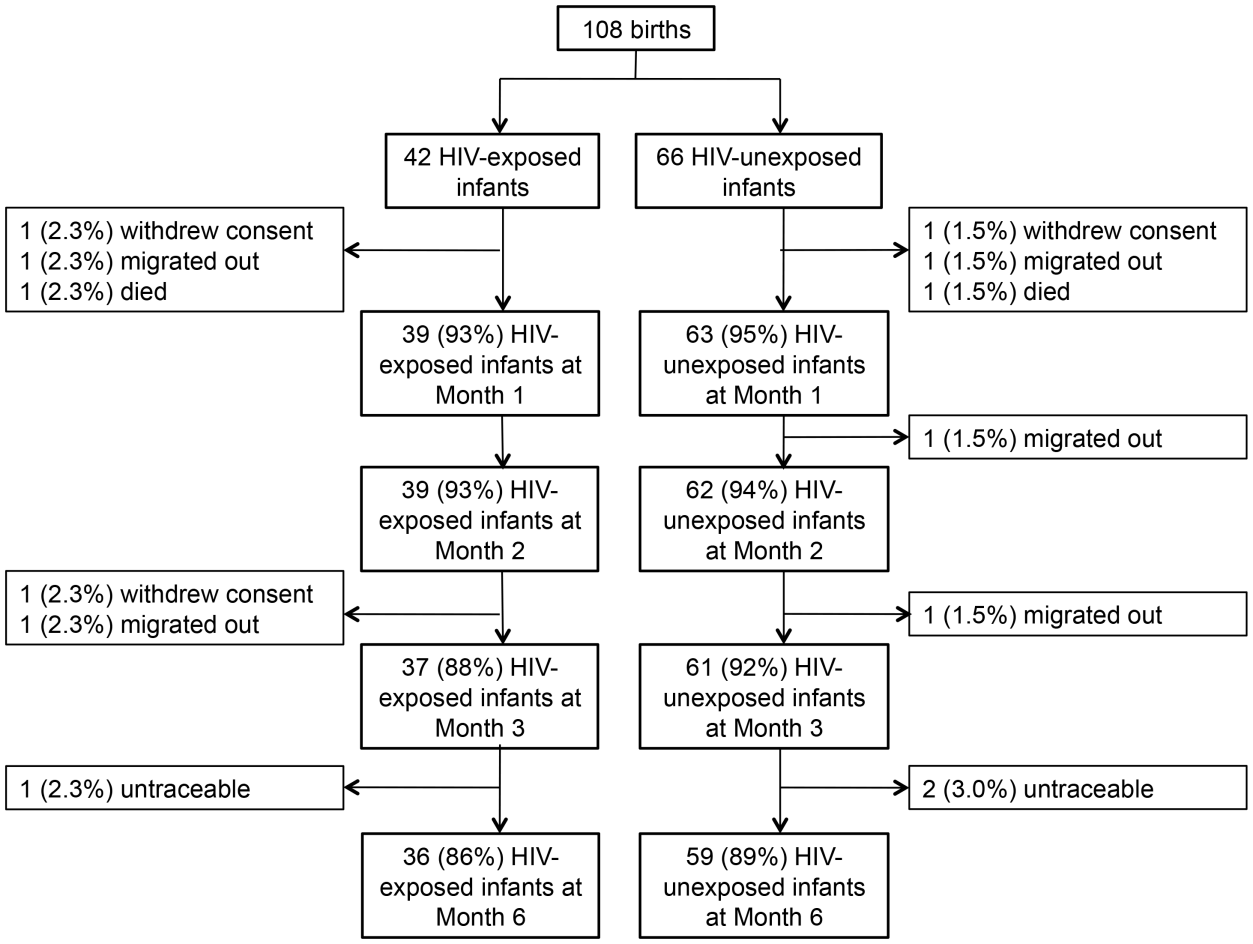
Study profile of infant cohort participants according to infant HIV-exposure.

All women were encouraged to deliver at KHC unless otherwise medically advised. As many women in this region do not deliver at health clinics, and preliminary research revealed that transportation expenses were the primary barriers to accessing healthcare [10], the study provided transportation compensation and other clinical expenses typically borne by mothers for delivery and follow-up visits. For women who delivered elsewhere, including home births, mothers and infants were requested to attend a follow-up clinic visit within three days of delivery. The study flow chart is summarized in Figure 1. If a mother-infant pair did not return for a regularly scheduled follow-up visit, a field worker traveled to their last known address to invite them to return to the clinic for a follow-up appointment.

At each follow-up, the research nurse, under supervision of the study coordinator, administered the Infant Feeding and Health Questionnaire to mothers. This questionnaire was designed to obtain data on a range of feeding, health, and environmental risk factors. Exclusive breastfeeding (EBF-WHO) was defined according to the WHO definition where “the infant receives breast milk (including expressed breast milk or breast milk from a wet nurse) and allows the infant to receive oral rehydration solution (ORS), drops, syrups (vitamins, minerals, medicines), but nothing else” [11]. Duration of EBF-WHO was defined as the time from birth until an infant first received food or liquids other than breast milk or medicines. Diarrhea was defined as loose or watery stools ≥ three times per day that represented a pattern atypical for that individual [2]. The questionnaire included: 1) infant nutrition: breastfeeding and complementary feeding practices; 2) mother-reported infant morbidity: cough, difficulty breathing, fever, convulsions, vomiting, skin rash, anorexia, unscheduled clinic/hospital visits, and episodes of diarrhea; and 3) environment: food security, using an index composed of questions relating to the mother's food consumption pattern, and sanitation and hygiene practices, such as hand-washing behavior, access to safe water, and toilet facilities. Infants exhibiting symptoms of illness were referred to the clinical officer at KHC for follow-up.

Anthropometric assessments were collected at each follow-up visit. Maternal height and weight were measured using a standard stadiometer (Health O Meter, Inc., Bridgeview, IL) to the nearest 0.2 kg and nearest 0.1 cm, respectively. Maternal mid-upper arm circumference (MUAC) and triceps skinfold thickness (TSF) were measured to the nearest 0.1 cm and 0.5 mm, respectively. Infant weight and length were measured using a calibrated digital infant scale (Seca 334 Digital Baby Scale) to the nearest 0.01 kg and a standard infant length board to the nearest 0.1 cm, respectively. Infant MUAC, TSF, and head circumference were measured to the nearest 0.1 cm, 0.5 mm, and 0.1 cm, respectively.

Active case detection was of interest so maternal and infant fecal samples were collected irrespective of self-reported intestinal symptoms at each follow-up visit. *Cryptosporidium* infection was detected using fresh stool samples that were stored in a cooler with ice packs for ≤5 hours before being transferred and stored at 4°C in the parasitology laboratory of the Tanzanian National Institute for Medical Research (NIMR), Mwanza Research Centre. Within 24 hours of collection, approximately 5 g of stool was mixed with 5 mL 10% v/v formalin and stored at 4°C until analysis. Presence of *Cryptosporidium* was confirmed using a modified Ziehl-Neelsen staining procedure [12], which is estimated to have a sensitivity ranging from 32 to 79% and a specificity ranging from 89 to 100% [13]–[15]. After staining, slides were examined by a single technician, without knowledge of participant clinical status, using a light microscope (Olympus model CX41RF) to detect *Cryptosporidium* oocysts and estimate oocyst burden. *Cryptosporidium* infection was defined as ≥1 oocyst detected in stained fecal smears. A second technician re-examined a sample (10%) of the slides and inter-observer agreement was 96%.

Data were analyzed in STATA10 (STATA Corporation, Texas, USA). Means of normally distributed continuous variables were compared using Student's *t*-test and proportions of categorical variables were compared using the χ^2^ test and Fisher's Exact test. Results were considered statistically significant at α = 0.05, two-sided. Univariate and multivariate logistic regression models were used to estimate the odds ratio (OR) and 95% confidence interval (95% CI) of *a priori* considered potential risk factors for infant *Cryptosporidium* infection (HIV-exposure, exclusive breastfeeding, maternal *Cryptosporidium* infection, and household factors, such as animal ownership, sanitation, wealth, and maternal education). This study is registered with ClinicalTrials.gov, number NCT01699841.

The sponsors (Cornell University and the National Science Foundation) were not involved in the design or oversight of the study. Members of the writing team had full access to the study data. The authors had final responsibility for the decision to submit for publication.

## Results

During the study period, 108 infants were born, and of these, six infants exited the study because of death, migration, or withdrawal of consent prior to the Month 1 study visit (Figure 1) and were not included in follow-up analyses. Birth anthropometrics were statistically different between HIV-exposed and HIV-unexposed infants (Table 1). A greater proportion of HIV-exposed infants had low birth weight (LBW; defined as birth weight <2500 g) compared to HIV-unexposed infants (HIV-exposed vs HIV-unexposed = 15 vs 3%, respectively; p = 0.026). Likewise, a greater proportion of HIV-exposed infants were stunted at birth (defined as birth length <44.7 cm) compared to HIV-unexposed infants (HIV-exposed vs HIV-unexposed = 18 vs 2%, respectively; p = 0.004). No HIV-exposed infant tested positive for HIV between birth and three months of age. Maternal and household characteristics did not differ based on HIV-status of the mother, other than marital status, where HIV-positive women were more likely to be divorced than HIV-negative women (HIV-positive vs HIV-negative = 21 vs 0%, respectively; p = 0.002).

**Table 1:** Anthropometric characteristics of infants at birth and baseline maternal characteristics.

INFANTS				
	All	HIV-exposed	HIV-unexposed	p value
**Sample Size**	102	39	63	
**Sex**				0.240
Male	52 (51%)	17 (44%)	35 (56%)	
Female	50 (49%)	22 (56%)	28 (44%)	
**Birth weight (kg)**				
Mean (SD)	3.2 (0.44)	3.1 (0.48)	3.3 (0.39)	0.028
Low birth weight (<2500 g)	8 (8%)	6 (15%)	2 (3%)	0.026
**Birth length (cm)**				
Mean (SD)	46.7 (0.22)	46.0 (0.41)	47.1 (0.22)	0.010
Stunted (<44.7 cm)	8 (8%)	7 (18%)	1 (2%)	0.003
**Birth MUAC (cm)**				
Mean (SD)	10.7 (0.11)	10.6 (0.19)	10.8 (0.13)	0.372
**Birth head circumference (cm)**				
Mean (SD)	34.5 (0.17)	34.2 (0.24)	34.7 (0.24)	0.127
Small head (<31.5 cm)	4 (4%)	1 (3%)	3 (5%)	0.578
**MOTHERS**				
	All	HIV-positive	HIV-negative	p value
**Age (years)**				
Mean (SD)	28.4 (5.9)	29.4 (6.0)	27.7 (5.8)	0.168
**CD4 cell count (cells/µL)**				
Median (IQR)		459 (330, 774)		
**Body mass index at Month 1**				
Mean (SD)	22.0 (2.6)	22.2 (2.7)	21.8 (2.5)	0.483
Underweight (<18.5 kg/m^2^)	8 (9%)	3 (8%)	5 (9%)	0.790
**Parity (number of children)**				
Mean (SD)	2.6 (1.7)	2.7 (1.9)	2.6 (1.6)	0.660
**Water**				0.342
Treats water	77 (76%)	27 (71%)	50 (79%)	
Does not treat water	24 (24%)	11 (29%)	13 (21%)	

SD = standard deviation; Stunting was defined using WHO growth standards where length-for-age z-score (LAZ) <−2 (44.7 cm for infants at birth) is considered stunted. Likewise, small head was defined as a birth head circumference <31.5 cm, which corresponds to a head circumference-for-age z-score<−2; MUAC = mid-upper arm circumference, there are currently no MUAC cut-off values for infants at birth [29]; IQR = interquartile range; Treats water = maternal report that the household takes measures to make water safe for drinking, i.e. boiling, filtration.

**Figure 2:**
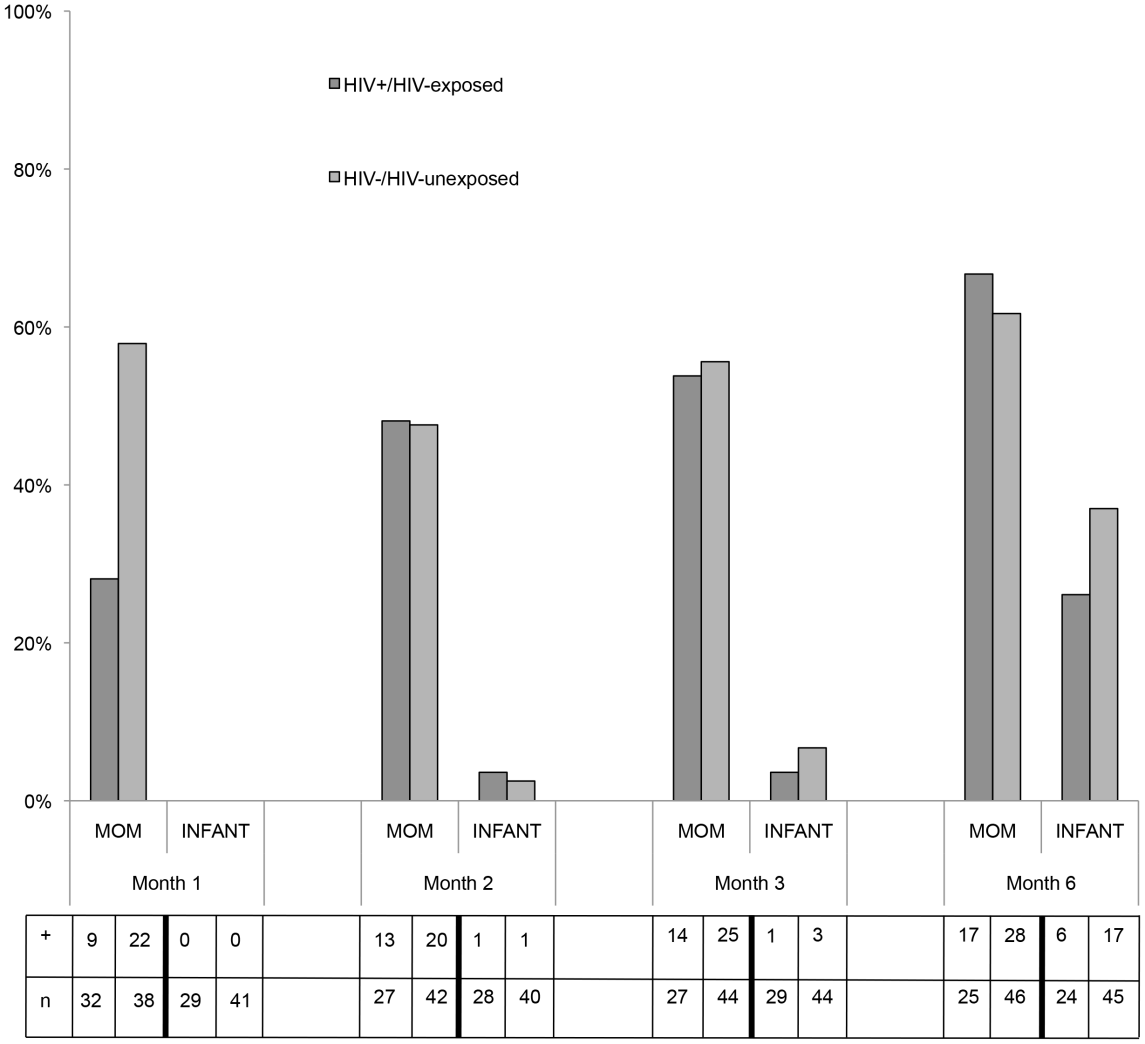
Prevalence of *Cryptosporidium* infection in mothers and infants according to HIV-status/exposure. +  = number of participants with evidence of *Cryptosporidium* infection; n = number of fecal samples analyzed. Note: the denominator increases across the study period for some groups due to missing data resulting from a missed appointment or failure to bring a fecal sample to the follow-up appointment.

The proportion of all mothers (HIV+ and HIV− combined) infected with *Cryptosporidium* ranged from a low of 44% (31/70) at Month 1 to a high of 63% (45/71) at Month 6 post-partum, and this proportion was not statistically different across time points. The majority of all mothers experienced *Cryptosporidium* infection at some point during the study follow-up period, with 82% experiencing *Cryptosporidium* infection at least once and 16% infected at every time point. Self-reported diarrhea was not related to *Cryptosporidium* infection and symptomatic infection ranged from a low of 0% at Month 3 to a high of 14% at Month 6. While the majority (60%) of mothers experienced self-recovery from *Cryptosporidium* infection between visits based on the presence/absence of oocysts in their feces, 15% of mothers who recovered later became re-infected on a subsequent visit. All infants remained free from *Cryptosporidium* infection until Month 2 and infection remained uncommon through Month 3. By Month 6, the increase in infection was dramatic with 33% (23/69) of infants exhibiting evidence of infection. Statistically significant differences in maternal *Cryptosporidium* prevalence based on HIV-serostatus were not evident, with the exception of the Month 1 study visit (p = 0.012) (Figure 2). There were no statistically significant differences in infant *Cryptosporidium* infection based on HIV-exposure (p = 0.284).

**Figure 3:**
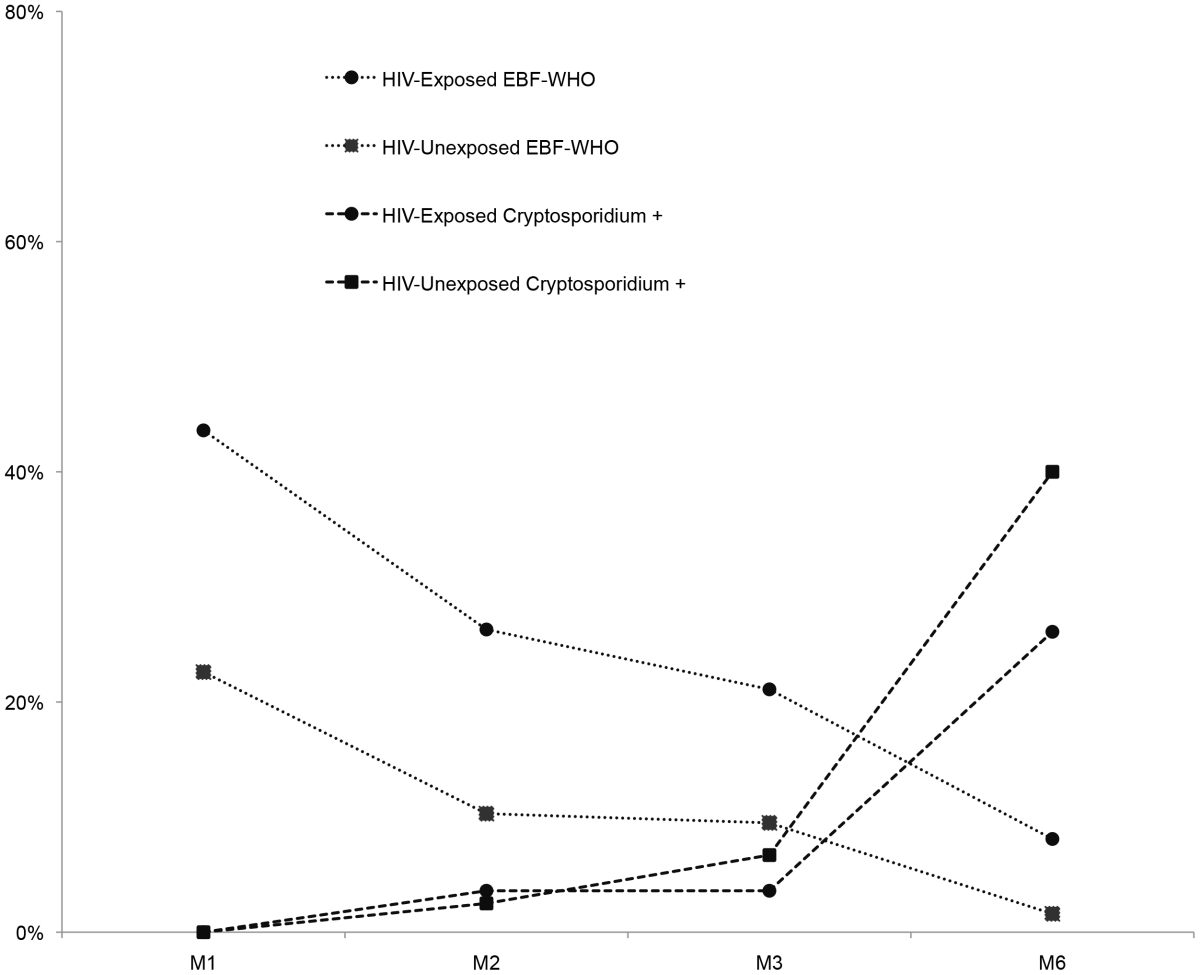
Proportion of infants exclusively breastfed (EBF) and proportion with *Cryptosporidium* infection according to HIV-exposure. M1 = Month 1; M2 = Month 2; M3 = Month 3; M6 = Month 6; EBF-WHO = WHO definition of exclusive breastfeeding.

**Figure 4:**
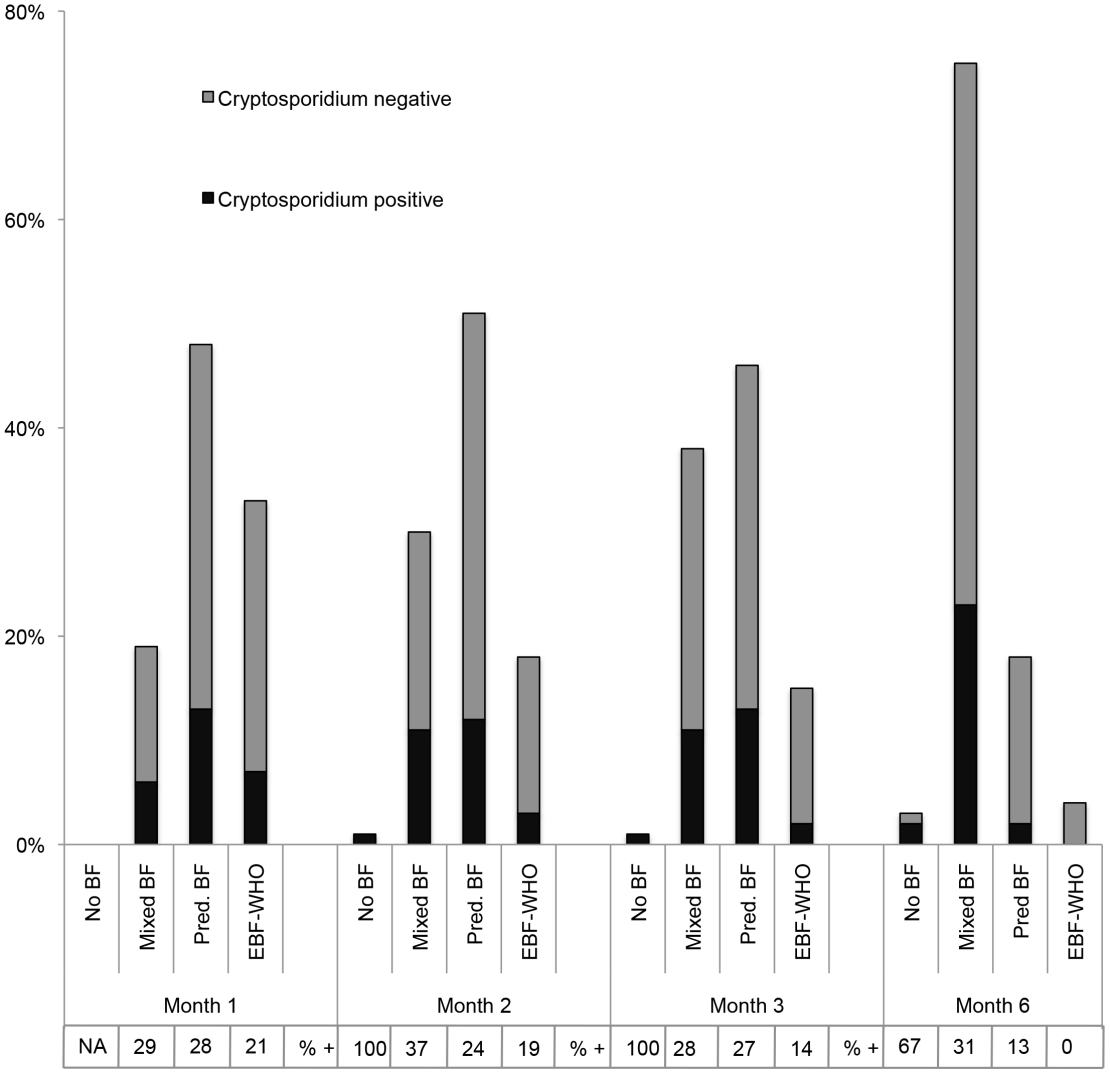
Proportion of infants infected with *Cryptosporidium* between 0 and 6 months according to status of breastfeeding practice. Cryptosporidium negative = *Cryptosporidium* was not detected in the feces of the infant during the study period; Cryptosporidium positive = *Cryptosporidium* was detected at least once during the study period; No BF (breastfeeding) = infant was not receiving any breast milk; Mixed BF = infant was receiving breast milk and other liquids and foods; Pred. BF = infant was receiving breast milk and locally prepared gripe water; EBF-WHO = WHO definition of exclusive breastfeeding.

As overall EBF-WHO declined, the proportion of infant *Cryptosporidium* infection increased (Figure 3). Post-partum, there was a higher proportion of HIV-positive mothers practicing EBF-WHO compared with HIV-negative mothers and this difference was statistically significant at both Month 1 (proportion HIV-positive vs HIV-negative: 44 vs. 23%, p = 0.03) and Month 2 (proportion HIV-positive vs HIV-negative: 26 vs. 10%, p = 0.04). Notably, of the four infants who continued EBF-WHO until six months, none had evidence of *Cryptosporidium* infection even though they were living in a *Cryptosporidium* environment as confirmed by evidence of maternal *Cryptosporidium* infection in all four cases. There was a pattern of lower proportion of *Cryptosporidium* infection in infants with a greater proportion of the diet consisting of breast milk (EBF-WHO vs. partial/no breastfeeding) and this was significant at Month 6 (p = 0.030) (Figure 4).

Neither maternal nor infant *Cryptosporidium* infection was associated with reported symptoms of infection that included diarrhea, anorexia, vomiting, and in mothers only, abdominal pain and nausea. Care-seeking behavior, operationalized as an unscheduled clinic or hospital visit, was uncommon for both mother (4%) and infant (8%) between each scheduled follow-up visit and was not associated with *Cryptosporidium* infection.

Table 2 summarizes the contribution of infant *Cryptosporidium* infection risk factors in this setting. In univariate analyses, only maternal *Cryptosporidium* infection at Month 1 (unadjusted OR = 3.18, 95% CI = 1.01 to 9.99) was associated with infant infection. While EBF-WHO was not significantly associated with lower odds of infant *Cryptosporidium* infection, there was a consistent trend between longer duration of EBF-WHO and lower infant infection. In the multivariate model, maternal hand washing prior to infant feeding was significantly associated with an increased likelihood of infant *Cryptosporidium* infection (adjusted OR = 5.02, 95% CI = 1.11 to 22.78). Maternal nutritional status, defined by body mass index (BMI) and MUAC, was not associated with maternal *Cryptosporidium* infection. Likewise, birth weight was not associated with infant *Cryptosporidium* infection nor was infant growth faltering up to six months a predictor of infant infection. Maternal food security index was negatively correlated with the practice of EBF-WHO at each visit; meaning that the more food secure a household, the less likely the infant was EBF-WHO. Similarly, the wealthier a household, the less likely the infant was EBF-WHO and the more educated a mother, the less likely the infant was EBF-WHO.

**Table 2:** Risk factors for infant *Cryptosporidium* infection between birth and six months.

	Infants (n)	Unadjusted OR (95% CI)	p value	Multivariate adjusted* OR (95% CI)	p value
**HIV**					
HIV-exposed	98	0.38 (0.14–1.07)	0.067	0.45 (0.10–1.98)	0.292
**Breastfeeding**					
EBF-WHO at Month 1	89	0.66 (0.23–1.90)	0.442		
EBF-WHO at Month 2	93	0.58 (0.15–2.22)	0.424		
EBF-WHO at Month 3	97	0.43 (0.09–2.09)	0.299		
PBF at Month 6	91	0.23 (0.05–1.09)	0.063	0.32 (0.05–2.08)	0.233
**Maternal ** ***Cryptosporidium***					
Crypto Month 1	69	3.18 (1.01–9.99)	0.047	3.40 (0.88–13.06)	0.075
Crypto Month 2	69	1.30 (0.47–3.63)	0.617		
Crypto Month 3	71	1.93 (0.63–5.89)	0.251		
Crypto Month 6	71	1.23 (0.42–3.58)	0.710		
Crypto any time	95	2.76 (0.58–13.12)	0.201		
**Household Factors**					
Owns Animals	98	0.80 (0.31–2.11)	0.654	0.75 (0.19–2.94)	0.676
Washes hands	98	1.72 (0.68–4·33)	0.249	5.02 (1.11–22.78)	0.036
Wealth	98	0.97 (0.31–3.00)	0.953	0.48 (0.09–2.44)	0.373
Maternal Literacy	98	1.51 (0.50–4.57)	0.466	0.76 (0.16–3.70)	0.735

*Adjusted for maternal HIV status (0 = negative; 1 = positive), PBF at Month 6 (0 = no breastfeeding or partial breastfeeding; 1 = predominant breastfeeding or exclusive breastfeeding), Maternal *Cryptosporidium* infection at Month 1 (0 = uninfected; 1 = infected), animal ownership (0 = no animals; 1 = owns animals), hand washing (0 = mother doesn't wash hands prior to infant feeding; 1 = mother washes hands prior to infant feeding), wealth (0 = lower 2 tertiles; 1 = top tertile), and maternal literacy (0 = mother cannot read; 1 = mother can read).

OR = odds ratio; CI = confidence interval; EBF-WHO = WHO definition of exclusive breastfeeding; PBF = exclusive or predominant breastfeeding; Washes hands = mother's self report of washing hands prior to feeding infant; Wealth = index (0–10) calculated by summing a categorical list of household possessions and then stratified into wealth (top tertile) vs. not wealthy (lower 2 tertiles); maternal literacy = mother's self-report that she can read.

## Discussion

This is the first report of maternal-infant *Cryptosporidium* infection in Sub-Saharan Africa and the prevalence of infection was high. Post-partum infection was detected at least once in the majority of women and, for many, on multiple occasions. The *Cryptosporidium* burden in infants increased dramatically between three and six months of age, a period that corresponds to changes in breast feeding practices. Our results indicate that young infants living in rural and semi-rural Tanzania are susceptible to *Cryptosporidium* infection in early infancy with approximately 1/3 of infants showing evidence of infection by six months of age.

This study confirms and extends the importance of *Cryptosporidium* infection in young infants reported in the GEMS study [2] that included both rural and urban settings. Our results are comparative to the findings of a sub-sample of young Tanzanian infants in urban, hospital-based studies where 25% of infants 0 to 6 months had evidence of either *G. lamblia* or *Cryptosporidium parvum* [16], though the burden of *Cryptosporidium parvum* was not individually reported. In studies conducted in the Tanzanian capital of Dar es Salaam, only 9% of children three months to nine years and 18.9% of children 0 to 60 months had evidence of *Cryptosporidium* infection [5], [16] and this may represent an urban-rural difference in young infant burden in Tanzania.

Previous studies in Tanzania of HIV-positive adults report a *Cryptosporidium* prevalence between 7 and 17% [5], [6] and HIV infection has been identified as a risk factor for *Cryptosporidium* and cryptosporidiosis in some studies [6], [17]–[20] but not others [2]. Maternal HIV infection did not appear strongly related to *Cryptosporidium* infection in our study and this may be explained in part because the majority of HIV-positive women were otherwise healthy and not severely immunocompromised based on their CD4 cell counts. Previous studies that identified HIV infection as a risk factor were primarily conducted in the pre-ARV era and greater immunosuppression may explain differences [6], [17], [20]. Likewise, HIV-exposure was not a significant risk factor for *Cryptosporidium* infection in infants and this might be due in part to more optimal feeding methods in the HIV-exposed infants due to infant feeding counseling for HIV-positive mothers. While HIV infection may not be a significant risk factor for infection in this setting, it remains relevant for the clinical management of cryptosporidiosis in immunocompromised individuals given the lack of effective *Cryptosporidium* treatment other than ARV's to improve HIV immunocompetency [21].

While maternal *Cryptosporidium* infection was associated with greater infant infection, previously (or even currently) infected mothers may also be providing protective passive immunity *in utero* or in breast milk. A recent study of Bangladeshi infants reported that protection from *Cryptosporidium* infection was associated with high anti-*Cryptosporidium* IgA in breast milk [22]. Despite possible passive immunity and/or risk elimination (from contaminated food/water), EBF-WHO was uncommon in our study population and was not sustained for the universally recommended duration of six months. In this study, using the WHO definition of EBF, only a third of mothers were practicing EBF-WHO at Month 1. Previous Tanzanian studies indicated much higher levels of “EBF” ranging from 49% within 3 days after birth [23], 90% at Month 1 [24], and 80% at Month 2 [25], but these large differences are likely due to the less strict non-WHO-EBF definitions and/or maternal recall methods used [24], [25]. Additionally, two of these studies included HIV-positive women only and HIV maternal care includes infant feeding counseling that is typically unavailable to HIV-negative mothers in this setting [24], [25]. Indeed, we found significantly higher rates of EBF-WHO in HIV-positive mothers and this may explain why infant HIV-exposure was associated with lower infant *Cryptosporidium* infections.

Globally, knowledge of the epidemiology of *Cryptosporidium* infection in early infancy is scarce and, in Tanzania, such data are unavailable. When the lack of prevalence data is combined with barriers to diagnosis, the disease rarely features on the clinician's diagnostic radar. This leads to a cycle that likely perpetuates the underestimation of the *Cryptosporidium* burden leading to an inappropriately lower global health and research priority. This cycle reinforces ineffective clinical and public health management of *Cryptosporidium*. In our study, maternal hand washing prior to infant feeding was counterintuitively associated with infant infection, although given the wide 95% confidence interval, we recommend caution in the interpretation of this finding. Previous studies have indicated that household sanitation and hygiene, including hand washing, were related to reduced *Cryptosporidium* infection [17]. Since *Cryptosporidium* has notoriously robust survival and transmissibility [26], [27], and mothers may wash their hands with contaminated water and then feed their children, our result is plausible in this setting. It may also be that the practice of hand washing is a proxy indicator for women who lived in more contaminated environments. Further research could include testing water sources and/or analysis of the species of *Cryptosporidium* in order to determine probable transmission routes of infection. Such investigations would help interpret this finding in relation to major public health messages related to hand washing in similar settings.

Our study had a number of limitations. First, at each follow-up visit, only one stool sample was collected from each mother and infant. Due to the intermittent shedding of *Cryptosporidium* oocysts, collection of a single stool sample may result in an underestimate of the true *Cryptosporidium* prevalence [28]. Additionally, our study used modified Ziehl-Neelsen staining, the most common diagnostic technique to detect the presence of *Cryptosporidium* oocysts in stool samples, however, the sensitivity and specificity of this method are not 100% leading to possible misclassification [13]. Lastly, our results may not be generalizable to other geographical settings due to urban/rural differences and geographical variation in *Cryptosporidium* contamination.

In conclusion, there is a high prevalence of infant and maternal *Cryptosporidium* infection in this setting. Public health interventions promoting EBF-WHO among all women, including HIV-negative mothers should be strengthened. Modeling the message of breast milk as an immunologically protective substance to prevent certain infectious diseases common in childhood may be effective in regions where there are high rates of vaccination coverage. Additionally, further research is needed to address efforts to minimize the maternal and environmental *Cryptosporidium* burden as a means of protecting young infants in the absence of effective vaccines, diagnostics, and treatment for early infancy cryptosporidiosis.

## Supporting Information

Checklist S1 **STROBE checklist.**
(DOC)

Table S1 **Baseline characteristics of mothers and households.**
(DOCX)
