# Supplementary material for: Cryptosporidium Prevalence and Risk Factors among Mothers and Infants 0 to 6 Months in Rural and Semi-Rural Northwest Tanzania: A Prospective Cohort Study
Source: PLoS Negl Trop Dis. 2014 Oct 2;8(10):e3072. doi: 10.1371/journal.pntd.0003072 (PMC4183438; doi:10.1371/journal.pntd.0003072)
Supplement: Table S1 — Baseline characteristics of mothers and households. (DOCX) [file pntd.0003072.s002.docx]

| **MOTHERS** | | | | |
| --- | --- | --- | --- | --- |
|  | All | HIV-positive | HIV-negative | p value |
| **Sample Size** | 102 | 39 | 63 |  |
| **Marital Status** |  |  |  | 0.002 |
| Single | 4 (4%) | 2 (5%) | 2 (3%) |  |
| Monogamous partner | 88 (86%) | 28 (72%) | 60 (95%) |  |
| Polygamous partner | 1 (1%) | 0 (0%) | 1 (2%) |  |
| Divorced/separated | 8 (8%) | 8 (21%) | 0 (0%) |  |
| Widowed | 1 (1%) | 1 (3%) | 0 (0%) |  |
| **Maternal Literacy** |  |  |  | 0.153 |
| Can Read | 76 (75%) | 26 (67%) | 50 (79%) |  |
| Cannot Read | 26 (25%) | 13 (33%) | 13 (21%) |  |
| **HOUSEHOLDS** | | | | |
|  | All | HIV-positive | HIV-negative | p value |
| **Household Size (people)** |  |  |  |  |
| Mean (SD) | 5.0 (2.6) | 5.1 (3.0) | 5.0 (2.4) | 0.839 |
| **Assets** |  |  |  |  |
| Animals |  |  |  | 0.718 |
| Yes | 37 (36%) | 15 (38%) | 22 (35%) |  |
| No | 65 (64%) | 24 (62%) | 41 (65%) |  |
| Electricity |  |  |  | 0.079 |
| Yes | 9 (9%) | 1 (3%) | 8 (13%) |  |
| No | 93 (91%) | 38 (97%) | 55 (87%) |  |
| Bicycle |  |  |  | 0.779 |
| Yes | 61 (60%) | 24 (62%) | 37 (59%) |  |
| No | 41 (40%) | 15 (38%) | 26 (41%) |  |
| Mobile Phone |  |  |  | 0.398 |
| Yes | 82 (80%) | 33 (85%) | 49 (78%) |  |
| No | 20 (20%) | 6 (15%) | 14 (22%) |  |

**Supplementary Table 1. Baseline characteristics of mothers and households.**
